# 2-(Phenyl­selenon­yl)pyridine

**DOI:** 10.1107/S1600536813029978

**Published:** 2013-11-20

**Authors:** Shivani Gulati, K. K. Bhasin, V. A. Potapov, Ekta Arora, Ray J. Butcher

**Affiliations:** a Department of Chemistry, D. A. V. College, Sector-10, Chandigarh, India; b Department of Chemistry & Centre of Advanced Studies in Chemistry, Panjab University, Chandigarh 160 014, India; c A.E. Favorsky Irkutsk Institute of Chemistry, 1 Favorsky Street, Irkutsk, RUS-664033, Russian Federation; d Department of Chemistry, Howard University, 525 College Street NW, Washington, DC 20059, USA

## Abstract

In the title compound, C_11_H_9_NO_2_Se, the pyridine and phenyl rings are almost perpendicular, with the dihedral angle between their mean planes being 79.16 (7)°. In the crystal, the mol­ecules pack so as to form ruffled sheets in the (110) plane connected by weak C—H⋯O inter­actions. In addition, there are weak π–π inter­actions between the mean planes of both the phenyl [centroid–centroid perpendicular distance of 3.591 (2) Å and slippage of 1.854 (2) Å] and pyridine rings [centroid–centroid perpendicular distance of 3.348 (2) Å and slippage of 1.854 (2) Å].

## Related literature

For the pharmacological activity of selenone derivatives, see: Abdel-Hafez & Hussein (2008 ▶); Zhao *et al.* (2012 ▶); Hassan *et al.* (2011 ▶); Bhabak *et al.* (2011 ▶). For the chemistry of selenium compounds bonded directly to pyridine, see: Bhasin *et al.* (2013 ▶). For the synthesis of pharmaceuticals, see: Nogueira & Rocha (2011 ▶). For the synthesis of perfumes, fine chemicals and polymers, see: Zeng *et al.* (2013 ▶).
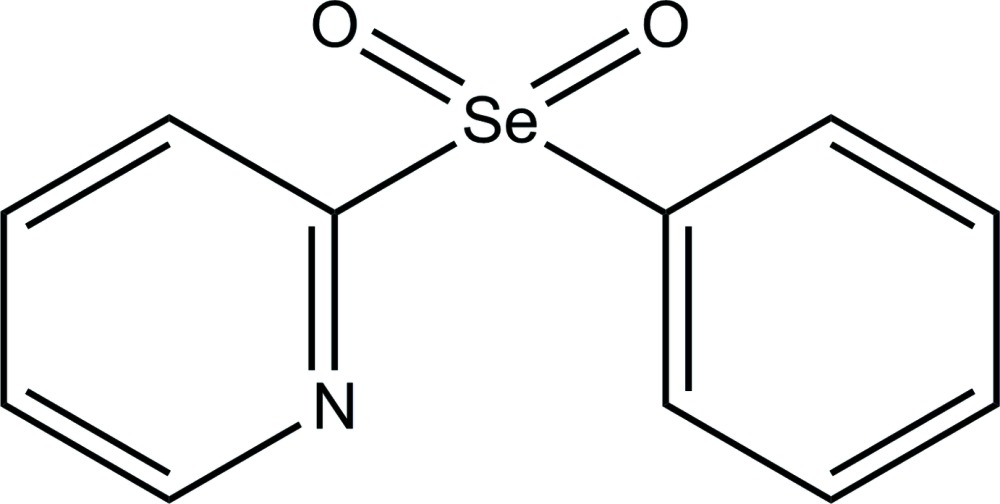


## Experimental

### 

#### Crystal data

C_11_H_9_NO_2_Se
*M* *_r_* = 266.15
Triclinic, 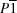

*a* = 6.1598 (5) Å
*b* = 7.7223 (6) Å
*c* = 11.4952 (7) Å
α = 80.683 (6)°
β = 83.494 (6)°
γ = 74.614 (7)°
*V* = 518.83 (7) Å^3^
*Z* = 2
Mo *K*α radiation
μ = 3.60 mm^−1^
*T* = 123 K
0.50 × 0.26 × 0.16 mm

#### Data collection

Agilent Xcalibur (Ruby, Gemini) diffractometer
Absorption correction: analytical (*CrysAlis PRO* and *CrysAlis RED*; Agilent, 2012 ▶) *T*_min_ = 0.383, *T*_max_ = 0.613
8688 measured reflections
5196 independent reflections
3965 reflections with *I* > 2σ(*I*)
*R*_int_ = 0.041

#### Refinement

*R*[*F* ^2^ > 2σ(*F* ^2^)] = 0.047
*wR*(*F* ^2^) = 0.090
*S* = 1.01
5196 reflections
136 parameters
H-atom parameters constrained
Δρ_max_ = 0.64 e Å^−3^
Δρ_min_ = −0.76 e Å^−3^

### 

Data collection: *CrysAlis PRO* (Agilent, 2012 ▶); cell refinement: *CrysAlis PRO*; data reduction: *CrysAlis PRO*; program(s) used to solve structure: *SHELXS97* (Sheldrick, 2008 ▶); program(s) used to refine structure: *SHELXL97* (Sheldrick, 2008 ▶); molecular graphics: *SHELXTL* (Sheldrick, 2008 ▶); software used to prepare material for publication: *SHELXTL*.

## Supplementary Material

Crystal structure: contains datablock(s) I, New_Global_Publ_Block. DOI: 10.1107/S1600536813029978/jj2170sup1.cif

Structure factors: contains datablock(s) I. DOI: 10.1107/S1600536813029978/jj2170Isup2.hkl

Supplementary material file. DOI: 10.1107/S1600536813029978/jj2170Isup3.cml

Additional supplementary materials:  crystallographic information; 3D view; checkCIF report

## Figures and Tables

**Table 1:** Hydrogen-bond geometry (Å, °)

*D*—H⋯*A*	*D*—H	H⋯*A*	*D*⋯*A*	*D*—H⋯*A*
C2*A*—H2*AA*⋯O1^i^	0.95	2.50	3.331 (3)	146
C4*A*—H4*AA*⋯O1^ii^	0.95	2.53	3.341 (3)	143
C5*A*—H5*AA*⋯O2^iii^	0.95	2.35	3.188 (3)	146

Symmetry codes: (i) 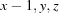; (ii) 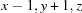; (iii) 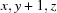.

**Fig. 1.**
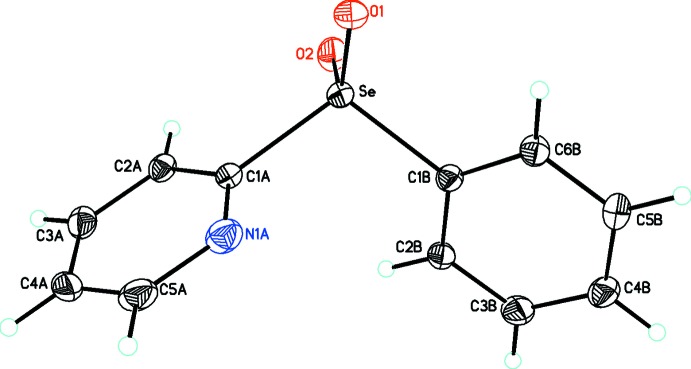
Molecular diagram of (I) illustrating the atom numbering scheme used. Thermal ellipsoids are at the 30% probability level.

**Fig. 2.**
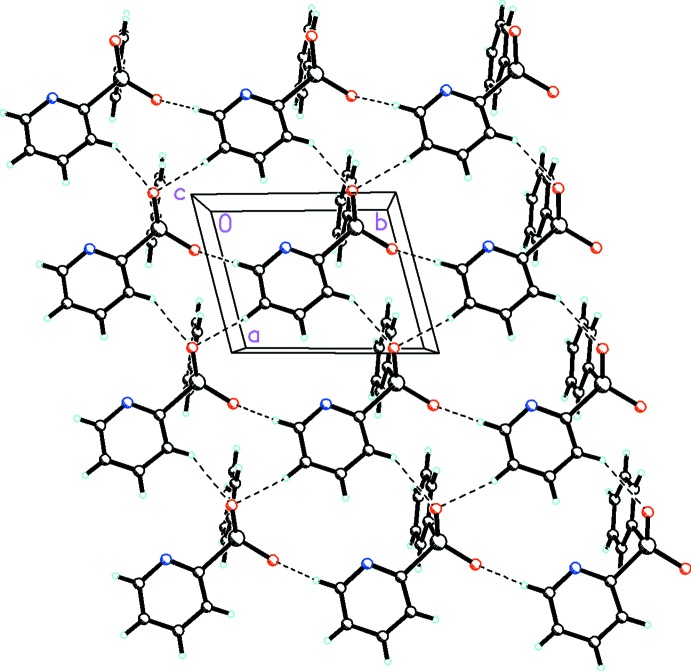
Fig, 2. Molecular packing for (I) viewed along the c axis. Dashed lines indicate the weak C—H···O intermolecular interactions forming ruffled sheets in the (1 1 0) plane.

**Fig. 3.**
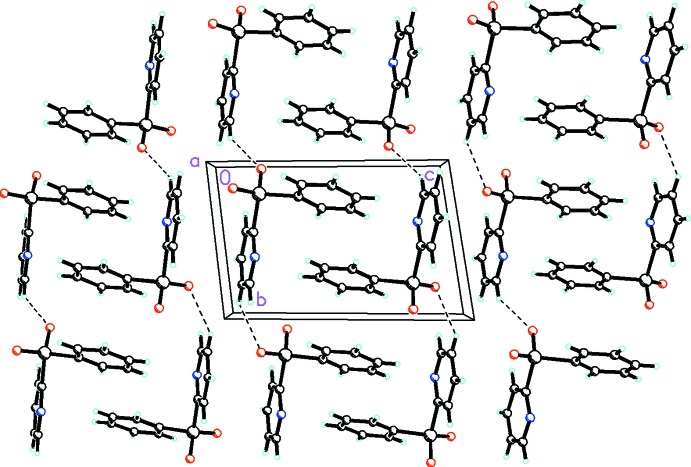
Molecular packing for (I) showing the π–π interactions between the mean planes of both the phenyl and pyridine rings.

## supplementary crystallographic information

### 1. Comment

Organochalcogen compounds, especially containing selenium have continued to attract attention of researchers in academia as anti-cancer (Zhao *et al*., 2012), anti-oxidant (Hassan *et al.*, 2011; Bhabak *et al*., 2011), anti-inflammatory and anti-allergic agents (Abdel-Hafez, & Hussein, 2008), and in industry because of their wide involvement as key intermediates for the synthesis of pharmaceuticals (Nogueira, & Rocha, 2011), perfumes, fine chemicals and polymers (Zeng *et al.*, 2013). Curiously, compared to alkyl, aryl and mixed alkyl aryl selenium compounds, the chemistry of selenium compounds bonded directly to pyridine has not yet been exploited extensively (Bhasin *et al.* 2013). In continuation of our ongoing program directed at the synthesis of novel organoselenium derivatives, we report here the synthesis and crystal structure of 2-(phenylselenonyl)pyridine.

In the title compound, C_11_H_9_NO_2_Se, (I), the pyridine and phenyl rings are almost perpendicular with the dihedral angle between the mean planes being 79.16 (7)° (Fig. 1). The molecules pack so as to form ruffled sheets in the (1 1 0) plane connected by weak C—H···O intermolecular interactions (Fig. 2). In addition there are weak π–π interactions between both the phenyl groups (*Cg*···*Cg* perpendicular distance of 3.591 (2) Å with slippage of 1.854 (2) Å [2 -*x*, -*y*, 1 - *z*]) and pyridine rings (*Cg*···*Cg* perpendicular distance of 3.348 (2) Å with slippage of 1.854 (2) Å [1 -*x*, 1 - *y*, -*z*]) (Fig. 3).

### 2. Experimental

A stirred solution of 2-(phenylseleninyl)pyridine (0.235 g, 1 mmol) in glacial acetic acid (10 ml) was treated with (0.550 g, 3.5 mmol) potassium permanganate in small amounts. The reaction mixture was allowed to stir for 3 h at room temperature. The progress of the reaction mixture was monitored by thin layer chromatography. After completion of the reaction, the reaction mixture was neutralized with excess of saturated solution of sodium bicarbonate and extracted with dichloromethane (4 *x* 25 ml). The combined organic extracts were washed with water and dried over anhydrous MgSO_4_. Dichloromethane was removed on a rota-evaporator that yielded a white powder. Single crystals of the compound suitable for XRD were prepared by dissolving the obtained white powder in a (1:1) mixture of CHCl_3_ and CCl_4_ followed by slow evaporation. Yield = 85%. *M*.p.= 453–455°K.

### 3. Refinement

All H atoms were placed in geometrically idealized positions and constrained to ride on their parent atoms with a C—H distance of 0.95 and *U*_iso_ (H) = 1.2*U*_eq_(C).

### Crystal data

**Table:**

C_11_H_9_NO_2_Se	*Z* = 2
*M**_r_* = 266.15	*F*(000) = 264
Triclinic, *P*1	*D*_x_ = 1.704 Mg m^−^^3^
Hall symbol: -P 1	Mo *K*α radiation, λ = 0.71073 Å
*a* = 6.1598 (5) Å	Cell parameters from 2941 reflections
*b* = 7.7223 (6) Å	θ = 3.1–37.5°
*c* = 11.4952 (7) Å	µ = 3.60 mm^−^^1^
α = 80.683 (6)°	*T* = 123 K
β = 83.494 (6)°	Triangular plate, colorless
γ = 74.614 (7)°	0.50 × 0.26 × 0.16 mm
*V* = 518.83 (7) Å^3^	

### Data collection

**Table:**

Agilent Xcalibur (Ruby, Gemini) diffractometer	5196 independent reflections
Radiation source: Enhance (Mo) X-ray Source	3965 reflections with *I* > 2σ(*I*)
Graphite monochromator	*R*_int_ = 0.041
Detector resolution: 10.5081 pixels mm^-1^	θ_max_ = 37.6°, θ_min_ = 3.1°
ω scans	*h* = −10→10
Absorption correction: analytical (*CrysAlis PRO* and *CrysAlis RED*; Agilent, 2012)	*k* = −12→13
*T*_min_ = 0.383, *T*_max_ = 0.613	*l* = −18→19
8688 measured reflections	

### Refinement

**Table:**

Refinement on *F*^2^	Primary atom site location: structure-invariant direct methods
Least-squares matrix: full	Secondary atom site location: difference Fourier map
*R*[*F*^2^ > 2σ(*F*^2^)] = 0.047	Hydrogen site location: inferred from neighbouring sites
*wR*(*F*^2^) = 0.090	H-atom parameters constrained
*S* = 1.01	*w* = 1/[σ^2^(*F*_o_^2^) + (0.0241*P*)^2^] where *P* = (*F*_o_^2^ + 2*F*_c_^2^)/3
5196 reflections	(Δ/σ)_max_ < 0.001
136 parameters	Δρ_max_ = 0.64 e Å^−^^3^
0 restraints	Δρ_min_ = −0.76 e Å^−^^3^

### Special details

**Table:**

Geometry. All e.s.d.'s (except the e.s.d. in the dihedral angle between two l.s. planes) are estimated using the full covariance matrix. The cell e.s.d.'s are taken into account individually in the estimation of e.s.d.'s in distances, angles and torsion angles; correlations between e.s.d.'s in cell parameters are only used when they are defined by crystal symmetry. An approximate (isotropic) treatment of cell e.s.d.'s is used for estimating e.s.d.'s involving l.s. planes.
Refinement. Refinement of *F*^2^ against ALL reflections. The weighted *R*-factor *wR* and goodness of fit *S* are based on *F*^2^, conventional *R*-factors *R* are based on *F*, with *F* set to zero for negative *F*^2^. The threshold expression of *F*^2^ > σ(*F*^2^) is used only for calculating *R*-factors(gt) *etc*. and is not relevant to the choice of reflections for refinement. *R*-factors based on *F*^2^ are statistically about twice as large as those based on *F*, and *R*- factors based on ALL data will be even larger.

### Fractional atomic coordinates and isotropic or equivalent isotropic displacement parameters (Å^2^)

**Table:**

	*x*	*y*	*z*	*U*_iso_*/*U*_eq_	
Se	0.80434 (4)	0.23043 (3)	0.224458 (18)	0.02645 (6)	
O1	1.0283 (3)	0.2030 (2)	0.13351 (14)	0.0370 (4)	
O2	0.6629 (3)	0.0770 (2)	0.24217 (15)	0.0380 (4)	
N1A	0.6710 (4)	0.6075 (3)	0.17338 (18)	0.0397 (5)	
C1A	0.5932 (3)	0.4583 (3)	0.17700 (17)	0.0247 (4)	
C2A	0.3857 (3)	0.4556 (3)	0.14449 (17)	0.0277 (4)	
H2AA	0.3426	0.3453	0.1487	0.033*	
C3A	0.2427 (4)	0.6216 (4)	0.10521 (19)	0.0358 (5)	
H3AA	0.0978	0.6274	0.0816	0.043*	
C4A	0.3131 (5)	0.7786 (3)	0.1008 (2)	0.0427 (6)	
H4AA	0.2166	0.8929	0.0741	0.051*	
C5A	0.5245 (5)	0.7685 (3)	0.1354 (2)	0.0453 (7)	
H5AA	0.5696	0.8777	0.1327	0.054*	
C1B	0.8984 (4)	0.2491 (3)	0.37495 (17)	0.0256 (4)	
C2B	0.7332 (4)	0.3166 (3)	0.45649 (18)	0.0298 (4)	
H2BA	0.5799	0.3593	0.4382	0.036*	
C3B	0.7969 (4)	0.3207 (4)	0.5661 (2)	0.0399 (6)	
H3BA	0.6853	0.3666	0.6251	0.048*	
C4B	1.0186 (4)	0.2598 (3)	0.5925 (2)	0.0369 (5)	
H4BA	1.0587	0.2646	0.6691	0.044*	
C5B	1.1842 (4)	0.1913 (3)	0.50800 (19)	0.0339 (5)	
H5BA	1.3373	0.1479	0.5267	0.041*	
C6B	1.1244 (4)	0.1864 (3)	0.39527 (18)	0.0290 (4)	
H6BA	1.2344	0.1419	0.3351	0.035*	

### Atomic displacement parameters (Å^2^)

**Table:**

	*U*^11^	*U*^22^	*U*^33^	*U*^12^	*U*^13^	*U*^23^
Se	0.02928 (11)	0.02457 (10)	0.02608 (11)	−0.00452 (8)	−0.00550 (7)	−0.00652 (8)
O1	0.0355 (9)	0.0416 (10)	0.0296 (8)	0.0001 (7)	0.0011 (6)	−0.0110 (7)
O2	0.0452 (10)	0.0285 (8)	0.0461 (10)	−0.0152 (7)	−0.0171 (8)	−0.0027 (7)
N1A	0.0512 (13)	0.0364 (11)	0.0364 (11)	−0.0169 (10)	−0.0067 (9)	−0.0069 (9)
C1A	0.0273 (9)	0.0254 (9)	0.0207 (8)	−0.0040 (7)	−0.0021 (7)	−0.0053 (7)
C2A	0.0274 (10)	0.0341 (11)	0.0219 (9)	−0.0085 (8)	0.0002 (7)	−0.0044 (8)
C3A	0.0293 (11)	0.0469 (14)	0.0254 (10)	−0.0010 (10)	0.0005 (8)	−0.0044 (10)
C4A	0.0578 (16)	0.0332 (12)	0.0253 (11)	0.0083 (11)	0.0003 (10)	−0.0052 (10)
C5A	0.078 (2)	0.0304 (12)	0.0326 (12)	−0.0202 (13)	−0.0063 (12)	−0.0063 (10)
C1B	0.0283 (10)	0.0251 (9)	0.0233 (9)	−0.0062 (8)	−0.0023 (7)	−0.0033 (8)
C2B	0.0265 (10)	0.0337 (11)	0.0262 (10)	−0.0013 (8)	−0.0007 (7)	−0.0070 (9)
C3B	0.0440 (14)	0.0417 (14)	0.0281 (11)	0.0002 (11)	0.0024 (9)	−0.0092 (10)
C4B	0.0481 (14)	0.0371 (12)	0.0248 (10)	−0.0079 (11)	−0.0055 (9)	−0.0051 (9)
C5B	0.0323 (11)	0.0383 (12)	0.0318 (11)	−0.0101 (10)	−0.0096 (9)	0.0003 (10)
C6B	0.0255 (10)	0.0334 (11)	0.0271 (10)	−0.0071 (8)	−0.0004 (7)	−0.0032 (9)

### Geometric parameters (Å, º)

**Table:**

Se—O1	1.6218 (16)	C5A—H5AA	0.9500
Se—O2	1.6234 (16)	C1B—C2B	1.359 (3)
Se—C1B	1.9240 (19)	C1B—C6B	1.381 (3)
Se—C1A	1.929 (2)	C2B—C3B	1.368 (3)
N1A—C1A	1.354 (3)	C2B—H2BA	0.9500
N1A—C5A	1.367 (3)	C3B—C4B	1.373 (3)
C1A—C2A	1.378 (3)	C3B—H3BA	0.9500
C2A—C3A	1.388 (3)	C4B—C5B	1.385 (3)
C2A—H2AA	0.9500	C4B—H4BA	0.9500
C3A—C4A	1.383 (4)	C5B—C6B	1.395 (3)
C3A—H3AA	0.9500	C5B—H5BA	0.9500
C4A—C5A	1.383 (4)	C6B—H6BA	0.9500
C4A—H4AA	0.9500		
			
O1—Se—O2	117.59 (9)	N1A—C5A—H5AA	118.8
O1—Se—C1B	106.80 (8)	C4A—C5A—H5AA	118.8
O2—Se—C1B	109.14 (8)	C2B—C1B—C6B	124.39 (19)
O1—Se—C1A	110.40 (9)	C2B—C1B—Se	116.78 (16)
O2—Se—C1A	106.21 (9)	C6B—C1B—Se	118.75 (15)
C1B—Se—C1A	106.17 (8)	C1B—C2B—C3B	117.2 (2)
C1A—N1A—C5A	115.3 (2)	C1B—C2B—H2BA	121.4
N1A—C1A—C2A	126.1 (2)	C3B—C2B—H2BA	121.4
N1A—C1A—Se	115.30 (16)	C2B—C3B—C4B	121.4 (2)
C2A—C1A—Se	118.53 (16)	C2B—C3B—H3BA	119.3
C1A—C2A—C3A	116.9 (2)	C4B—C3B—H3BA	119.3
C1A—C2A—H2AA	121.6	C3B—C4B—C5B	120.3 (2)
C3A—C2A—H2AA	121.6	C3B—C4B—H4BA	119.8
C4A—C3A—C2A	119.4 (2)	C5B—C4B—H4BA	119.8
C4A—C3A—H3AA	120.3	C4B—C5B—C6B	119.5 (2)
C2A—C3A—H3AA	120.3	C4B—C5B—H5BA	120.3
C3A—C4A—C5A	119.8 (2)	C6B—C5B—H5BA	120.3
C3A—C4A—H4AA	120.1	C1B—C6B—C5B	117.1 (2)
C5A—C4A—H4AA	120.1	C1B—C6B—H6BA	121.4
N1A—C5A—C4A	122.5 (2)	C5B—C6B—H6BA	121.4
			
C5A—N1A—C1A—C2A	1.2 (3)	O1—Se—C1B—C2B	164.23 (17)
C5A—N1A—C1A—Se	177.60 (16)	O2—Se—C1B—C2B	−67.68 (19)
O1—Se—C1A—N1A	−60.19 (17)	C1A—Se—C1B—C2B	46.41 (19)
O2—Se—C1A—N1A	171.30 (15)	O1—Se—C1B—C6B	−18.7 (2)
C1B—Se—C1A—N1A	55.21 (17)	O2—Se—C1B—C6B	109.38 (18)
O1—Se—C1A—C2A	116.53 (16)	C1A—Se—C1B—C6B	−136.53 (18)
O2—Se—C1A—C2A	−11.98 (18)	C6B—C1B—C2B—C3B	−0.6 (4)
C1B—Se—C1A—C2A	−128.08 (16)	Se—C1B—C2B—C3B	176.31 (18)
N1A—C1A—C2A—C3A	−0.5 (3)	C1B—C2B—C3B—C4B	0.3 (4)
Se—C1A—C2A—C3A	−176.84 (14)	C2B—C3B—C4B—C5B	−0.3 (4)
C1A—C2A—C3A—C4A	−0.1 (3)	C3B—C4B—C5B—C6B	0.7 (4)
C2A—C3A—C4A—C5A	0.0 (3)	C2B—C1B—C6B—C5B	0.9 (3)
C1A—N1A—C5A—C4A	−1.2 (3)	Se—C1B—C6B—C5B	−175.90 (16)
C3A—C4A—C5A—N1A	0.7 (4)	C4B—C5B—C6B—C1B	−0.9 (3)

### Hydrogen-bond geometry (Å, º)

**Table:**

*D*—H···*A*	*D*—H	H···*A*	*D*···*A*	*D*—H···*A*
C2*A*—H2*AA*···O1^i^	0.95	2.50	3.331 (3)	146
C4*A*—H4*AA*···O1^ii^	0.95	2.53	3.341 (3)	143
C5*A*—H5*AA*···O2^iii^	0.95	2.35	3.188 (3)	146

Symmetry codes: (i) *x*−1, *y*, *z*; (ii) *x*−1, *y*+1, *z*; (iii) *x*, *y*+1, *z*.
